# MALDI-TOF MS and Machine Learning Explanations for the Detection of SARS-CoV‑2 Infection in Human Plasma: Fingerprints as a Strategy for Risk Assessment

**DOI:** 10.1021/acsomega.5c04593

**Published:** 2025-09-08

**Authors:** Meritxell Deulofeu, Esteban García-Cuesta, Eladia María Peña-Méndez, José Elías Conde-González, Orlando Jiménez-Romero, Enrique Verdú, Maria Teresa Serrando, Victoria Salvadó, Pere Boadas-Vaello

**Affiliations:** † Research Group of Clinical Anatomy, Embryology and Neuroscience (NEOMA), Department of Medical Sciences, 16738 University of Girona, Girona 17003, Catalonia, Spain; ‡ Department of Artificial Intelligence, 16771 Universidad Politécnica de Madrid, Madrid 28223, Spain; § Department of Chemistry, Analytical Chemistry Division, Faculty of Sciences, 16749 University of La Laguna, San Cristóbal de La Laguna, Tenerife 38204, Spain; ∥ ICS-IAS Girona Clinical Laboratory, Santa Caterina Hospital, Parc Sanitari Martí i Julià, Salt 17190, Catalonia, Spain; ⊥ Department of Chemistry, Faculty of Science, University of Girona, Girona 17071, Catalonia, Spain

## Abstract

The COVID-19 pandemic proved to be a major public health challenge that had an enormously disruptive effect on the operational management of hospitals. It became especially important to find both diagnostic and prognostic methods for risk severity evaluation. Here, a MALDI-TOF MS method for the profiling of plasma samples combined with machine learning (ML) and its explanations was developed to identify SARS-CoV-2 infection while also allowing for the classification of patients by the severity of the disease. A prospective study of the most important *m*/*z* values that can be used as biomarkers using the SHAP state of the art ML explicability technique was also studied. The fingerprint data-analysis strategy is concerned with pattern expression in serum samples, providing information about SARS-CoV-2. The trained model is found to have a significant power of discrimination between controls and COVID-19 patients, controls and patients in the ICU, and controls and patients who had been in the ICU, and so, a spectral signature can be identified to separate and identify these cases. Moreover, there were differences in the spectral signatures between patients who were in the ICU and those who were not admitted to the ICU or had left the ICU. In conclusion, MALDI-TOF MS and advanced ML algorithms demonstrated remarkable discriminatory power between controls and those diagnosed with COVID-19/ICU/Post-ICU conditions. Also, it provides a valuable tool for stratifying patients based on their severity symptoms. Finally, a set of potential biomarkers that play a crucial role in the discrimination were identified.

## Graphical abstract

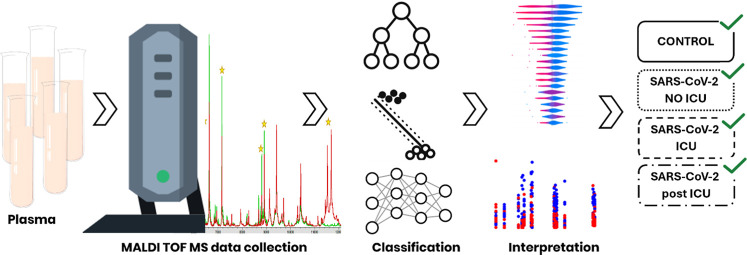

## Introduction

Since the end of 2019, the COVID-19 pandemic has proved to be an enormous challenge for human healthcare and was for many months a fundamental obstacle to economic development around the world. In terms of hospital management, the pandemic presented enormous difficulties that will in most cases have been a completely new experience. Any scientific assistance that can be provided to hospitals in allowing them to prioritize those patients that require more immediate or intensive attention can be of significant benefit only in helping hospitals to meet this challenge. The situation has been further complicated by the emergence of new variants of SARS-CoV-2, some of which are highly contagious. This increased the need for simpler, fast, cheap, and accurate diagnostic and prognostic methods for earlier detection and evaluation of the severity of the risk.^1^ The gold standard for the detection of the SARS-CoV-2 is quantitative real-time RT-qPCR even if the immunological assays using respiratory samples are also currently used for the diagnosis of this disease.^2,3^ Matrix- assisted laser desorption ionization (MALDI)–time-of-flight (TOF)–mass spectrometry (MALDI-TOF MS) is a bioanalytical technique that is used in the identification of cultured bacteria, viruses, and fungi in clinical microbiology.^4–6^ Since 2020, different methodologies based on MALDI-TOF MS have been proposed as an alternative to PCR-based strategies given that they are rapid, accurate, and cost-effective.^2,7,8^

The fingerprints of different biological samples, which represent the ionizable protein components that may correspond to a pathogen or disease state, are obtained by MALDI-TOF MS, and the application of artificial intelligence methods such as machine learning (ML) or artificial neural networks can then be used to differentiate samples (e.g., control vs disease samples).^7,9^ The above-mentioned applications are based on the matching of sample MS spectra against reference spectra, and in the second one, the authors also use X-AI techniques to provide interpretability of the model. Methods based on MALDI-TOF analyses of human biofluids have been proposed for the diagnosis of myeloma and other cancers and other diseases.^6,9^

In previous research, several methods have been developed that require only minimal sample preparation and achieve high performance of over 90% in terms of accuracy, sensitivity, and specificity without the need to identify biomarkers.^10–13^

The proteomic and metabolomic analysis of sera samples performed in several studies^14–22^ have demonstrated the feasibility of using sera profiles for the diagnosis of COVID-19 and to differentiate expressed factors that can be correlated with disease severity. Quantitative proteomic analysis of plasma allowed the identification of the changes in the plasma proteome signature due to the host response to SARS-CoV-2 infection,^15^ whereas proteins associated with blood coagulation (D-dimer), cell damage (lactate dehydrogenase), and the inflammatory response (for example, C-reactive protein) have already been identified as possible predictors of COVID-19 severity and mortality.^16^ The changes in abundance of a set of proteins identified in serum samples from Spanish COVID-19 patients correlate with patient age and disease severity.^17^ A proteomic approach using data-independent acquisition mass spectrometry identified 27 proteins that were differentially expressed between severely ill COVID-19 patients with adverse and favorable prognoses.^18^

Shen et al.^14^ showed that it is possible to classify severe cases using serum proteins and metabolite biomarkers and to predict the progression of the illness. The combination of mass spectrometry with ML allowed the selection and identification of 19 molecules related to the disease’s pathophysiology providing tools for risk assessment in patient management in hospitals.^19^ Peptidome-based studies using serum from patients and high-throughput spectrometric techniques promise to be valuable for the identification of COVID-19-associated biomarkers. To this end, various ML algorithms were applied in MALDI-TOF-based serum peptidome profiling to build classification models using 25 feature peaks, 15 of which were identified by proteomic analysis.^20^ A similar approach was used for the identification of a plasma proteomic signature obtained from high- (hospitalized) versus low- (outpatients) risk patients with COVID-19, showing that serum amyloid A1 (SAA1) and A2 proteoforms were differentially expressed between the two groups.^21^ Serum amyloid A2 protein was identified as one of the major biomarkers of severe COVID-19 patients by Gomilla et al.,^22^ who also demonstrated that MALDI-TOF generated peptidome profiles can be used as clinical classifiers.

Recently, a review published into metabolomic studies on COVID-19 severity highlighted that exhaled air, saliva, plasma, and urine are all now being used to identify diagnostic and prognostic biomarkers.^23^ However, proteomic and metabolomic analyses of serum that were performed after MALDI-TOF analyses to identify the changes in proteins and metabolites in the plasma are time-consuming and require specialized personnel, and other MS-based techniques generally coupled to liquid chromatography are not typically available in hospital settings.

The present study aims to develop a simple, fast, and robust method based on MALDI-TOF MS profiling of plasma samples combined with artificial intelligence to identify SARS-CoV-2 infection, which will allow the classification of patients by the severity of the disease. Three specific questions are addressed: (1) can we differentiate between samples of people infected or not with SARS-CoV-2?; (2) can we distinguish between patients who have needed treatment in the ICU and those who have not?; and (3) can we distinguish between patients who have recovered from the disease and those who remain hospitalized? Furthermore, we aim to observe whether age stands out as a criterion for a worse prognosis. A prospective study of the most important *m*/*z* values that may be used as biomarkers using the SHAP state of the art explicability technique is also presented.

## Results and Discussion

### Differences in the Mass Spectra of the Plasma Samples between COVID-19 and the Control Groups

To make an initial verification of the ability of MALDI-TOF MS to distinguish between Control and Positive COVID-19 samples, a statistical analysis of the MALDI-TOF MS *m*/*z* fingerprints was performed. The pattern in the mass spectra of control samples remained relatively stable irrespective of the batch, unlike those corresponding to the COVID-19 positive samples (Figure 1).

**Figure 1:**
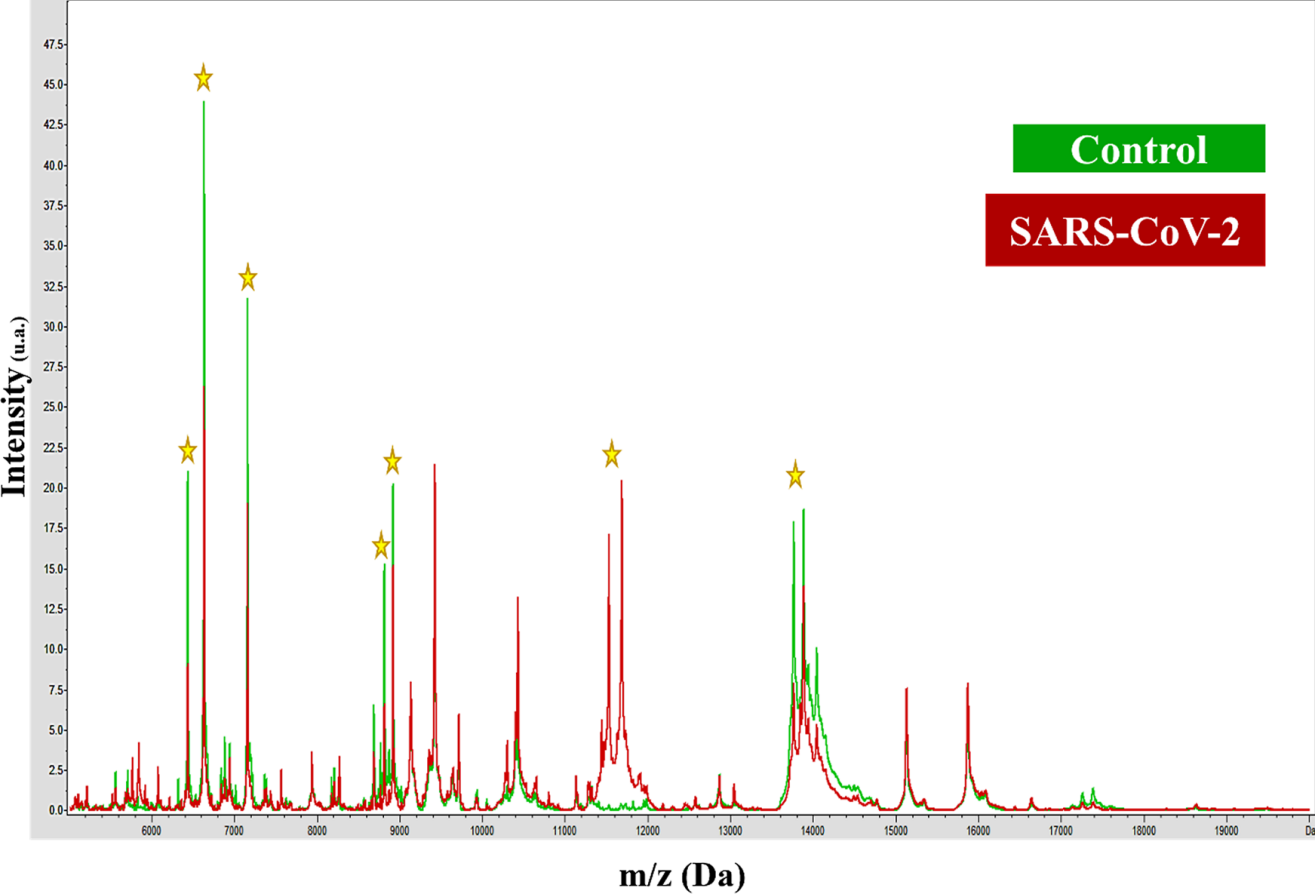
Representative MALDI-TOF mass spectra of plasma from control and SARS-CoV-2-positive samples. The yellow marks the most significant signals for differentiation.

To understand how the information is distributed across the fingerprints (*m*/*z* predictors), the current study attempted to identify characteristics that permit the differentiation of plasma samples leading to different pattern-like mass spectra that are equivalent to MALDI-TOF-MS fingerprints. The mass spectra of the two groups presented differences both in the intensity of signals and the *m*/*z* values in some regions of the spectra, the most obvious of which are located at the *m*/*z* range of 6 to 15 kDa. Study of the mass spectra shows two *m*/*z* regions that present the most significant differences (marked with yellow stars in Figure 1) between the control and positive SARS-CoV-2 samples. The differences found were more significant in the 11,000–12,000 and 13,800–14,800 mass- to-charge (*m*/*z*) regions. The region between 10 and 15 kDa, and especially the 11,300–11,700 region, was identified when ML was applied to classify the groups as being suitable for the differentiation of high and low risk COVID-19 patients, and four proteins, including platelet basic protein, immunoglobulin lambda variable 4–69 (IGLV4-69), and serum amyloid A-1 and A-2, were identified as being up-regulated in the high-risk group.^21,22^ Comparison of the individual *m*/*z* values of the two groups reveals significant differences in the mass spectra, which were further analyzed by Box and Whisker plots. These plots show the median value as the center bar in the box, and the first (Q1) and third (Q3) quartiles as the box boundaries, with whiskers and outliers extending to either the Q1–1.5IQR and the Q3 + 1.5IQR (IQR: Inter Quartile Range). As an example, the plots shown in Figure 2 illustrate the presence of differences in signal levels for four selected *m*/*z* values.

**Figure 2:**
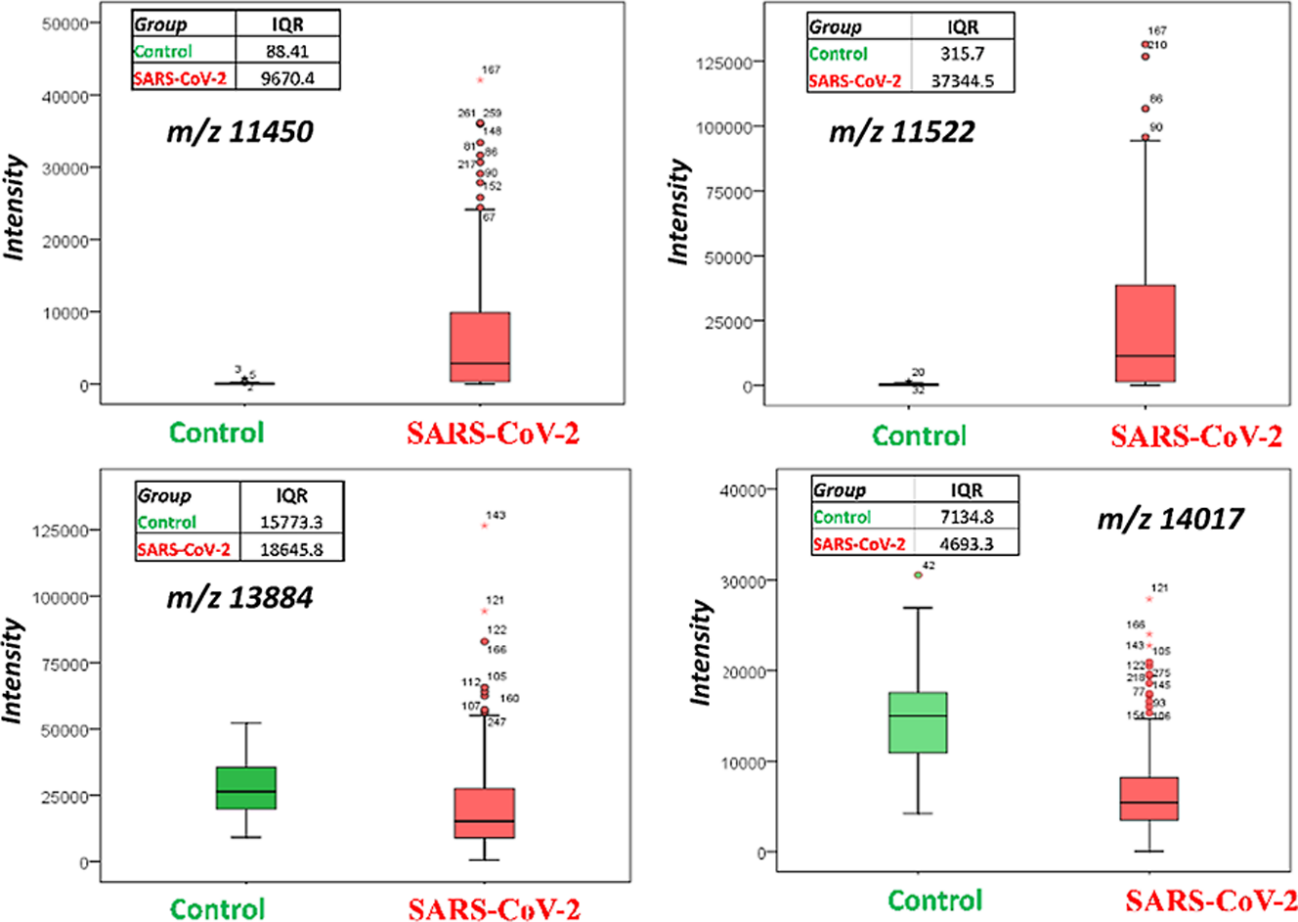
Box and Whisker plots of selected *m*/*z* values for control and SARS-CoV-2 samples.

The intensities of *m*/*z* values 11,450 and 11,522 were clearly different between the COVID-19 sample and the control group. These *m*/*z* peaks can be associated with proteoforms of SAA1 and SAA2. Lazari et al. 2021 reported that the region between 10 and 15 kDa is suitable for the differentiation between high- and low risk patients and identified truncated fragments originating from SAA1 and A2 (SSA2) in the 11,400–11,700 regions.^21^ In the case of the *m*/*z* values of 13,884 and 14,017, the intensities of these peaks for the COVID-19 group are lower than those of the control group. The first *m*/*z* may correspond to pro-platelet basic protein (PPBP/CXCL7, 13,894 Da), a platelet-expressing chemokine that is downregulated in severe COVID-19 patients,^14^ and that was identified as a potential biomarker of critical worsening (patients requiring intubation), which is a finding shared by other studies.^24,25^ The association of 14,017 to a specific biomolecule is difficult as it can be assigned to human protein MAX (14,033.17) or to mannose-binding lectin protein (MBL, 14,092). However, it has been demonstrated that the lectin pathway is activated during SARS-CoV-2 infection, with higher activation in more severe cases of COVID-19.^26^ Figure 3 contains a selection of Box and Whisker plots showing the differences between the control and SARS-CoV-2 positive groups and between patients by whether or not they had been admitted to the ICU.

**Figure 3:**
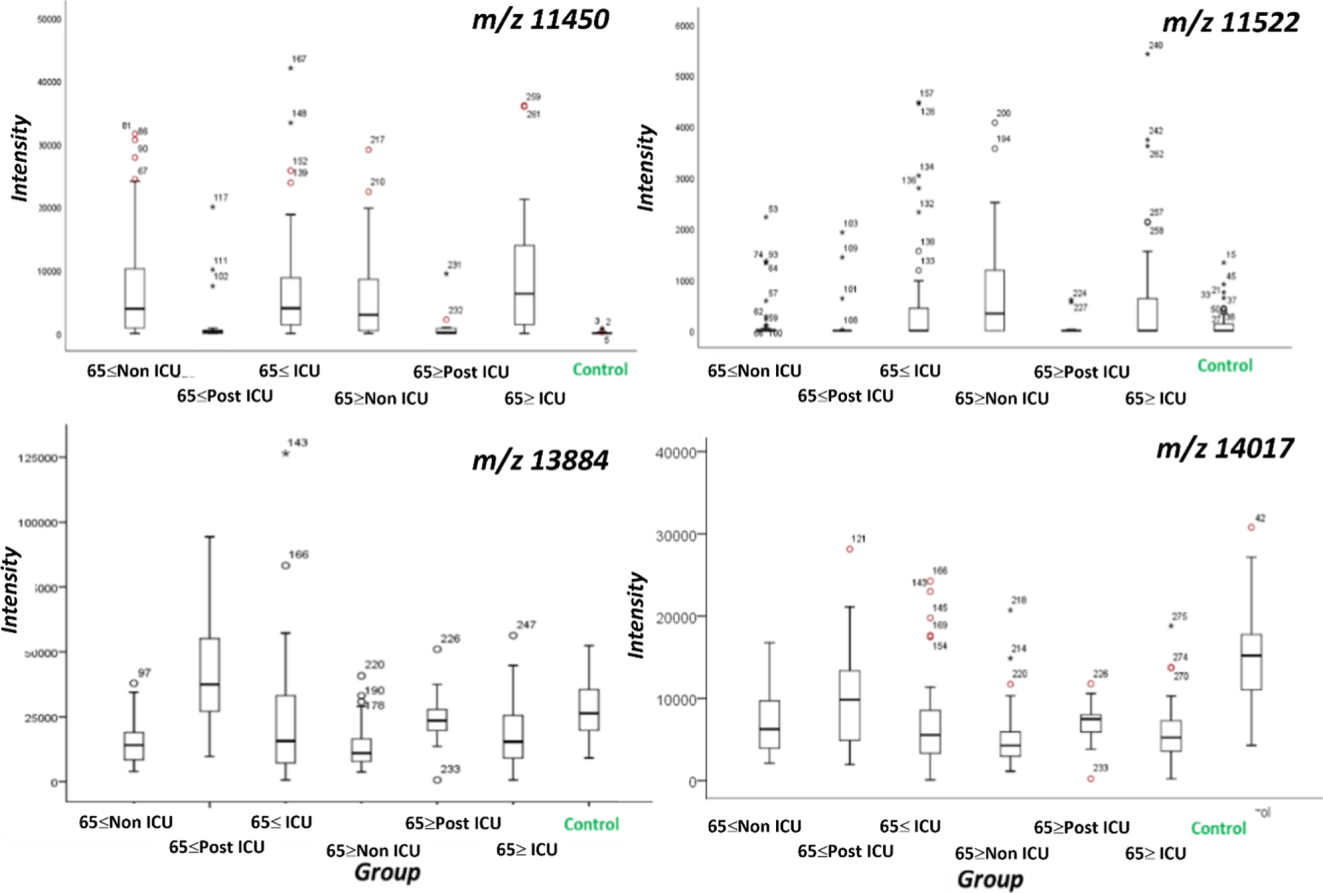
Box and Whisker plots differences in patterns for selected *m*/*z* values between the different groups.

### Machine Learning Results

The fingerprint data-analysis strategy is concerned with pattern expression in serum samples, providing information about SARS-CoV-2 in humans. In addition to investigating the information shown above, we were interested in evaluating the predictive value of mass spectra fingerprints by comparison with readily available predictors of prognosis in humans (ICU stay, no stay at ICU, and age). Values of the receiver operating characteristic curve (AUROC), which is one of the most important evaluation metrics for checking any classification model’s performance, were accepted whenever they were higher than 0.7. Table 1 shows the best results for each classification task in bold. All the hypotheses, except for “ICU vs. Post_ICU” (patients in the Intensive Care Unit vs patients who have been discharged from the Intensive Care Unit) and “ICU ≥65 vs. ICU <65”, have an AUROC above 0.7. That means that the ML models are able to learn, at some extend, a pattern that allows discrimination between the two presented classes. For “COVID-19 vs. Control” (patients who have COVID-19 vs those who do not), “ICU vs. Control”, and “Post_ICU vs. Control”, these values are above 0.95 that is an outstanding performance according to the literature. The 0.75 AUROC value in the “ICU vs. Non_ICU” (patients in the Intensive Care Unit vs those that have COVID-19 but who were not admitted to the Intensive Care Unit at any point) experiment is particularly remarkable given that is clearly above the no discrimination value of 0.5 and so it can be considered that the model has an acceptable discrimination power. Given this, we can conclude that we can discriminate “COVID-19 vs. Control”, “ICU vs. Control”, “Post-ICU vs. Control” and, therefore, there is likely to be a spectral signature that can be used to separate and identify these cases. Moreover, the power of discrimination for “ICU vs. Non_ICU, ICU & Post_ICU vs. Non_ICU & Control” with an AUROC values of 0.75 and 0.77 is acceptable. This also means that there are differences in the spectral signatures between patients that are in the ICU and those that have COVID-19 but are not in the ICU. Finally, we observe that “ICU vs. Non_ICU & Control” also has an AUROC value of 0.78, but it basically reinforces the previous hypothesis of “ICU vs. Non_ICU” while adding control instances that are easily discriminated as we prove in the “ICU vs. Control” experiment and, therefore, slightly improves the results but does not provide new insights. In the following experiment, we addressed the problem of the interpretation of the models created by making use of the full mass spectrum information. We did not find a clear discriminative model for people in the ICU between patients younger than 65 and those who are older than 65. This reinforces the hypothesis that there are no virus-related changes to the plasma composition due to age. However, it has been found in proteomic studies by another group that the alteration in the abundance of some serum proteins with an increase in the disease severity might correlate with the age of the hospitalized patients.^17^ Demichev et al. (2021) also observed that a number of markers, including proteins and clinical laboratory markers, increase with age in COVID-19 patients and have identified proteins that are up- or downregulated in older patients in comparison with younger patients within the same level of severity.^27^ These proteins correlated only with age in COVID-19 patients and include markers involved in inflammation such as SAA1 and A2 proteoforms.

**Table 1:** Support Vector Machine (SVM), XGBOOST, and MLP Test Results (Bold Values Represent the Best Results Obtained Whenever Its AUROC Value Is Higher Than 0.7, Which Is Considered Acceptable)

	SVM				XGBOOST				MLP			
experiment	accuracy	sensitivity	specificity	AUROC	accuracy	sensitivity	specificity	AUROC	accuracy	sensitivity	specificity	AUROC
COVID-19 vs control	0.97 ± 0.03	0.97 ± 0.04	0.98 ± 0.03	0.97 ± 0.03	0.94 ± 0.03	0.98 ± 0.02	0.95 ± 0.03	0.88 ± 0.07	0.98 ± 0.02	0.99 ± 0.02	0.99 ± 0.02	0.96 ± 0.06
ICU vs control	0.96 ± 0.04	0.97 ± 0.04	0.97 ± 0.04	0.96 ± 0.04	0.93 ± 0.03	093 ± 0.04	0.96 ± 0.03	0.93 ± 0.03	0.96 ± 0.03	0.97 ± 0.04	0.97 ± 0.06	0.96 ± 0.03
ICU vs No_ICU	0.75 ± 0.04	0.71 ± 0.09	0.77 ± 0.12	0.75 ± 0.04	0.73 ± 0.07	0.75 ± 0.07	0.72 ± 0.10	0.73 ± 0.07	0.74 ± 0.08	0.73 ± 0.13	0.72 ± 0.12	0.74 ± 0.08
ICU ≥65 vs No_ICU ≥65	0.73 ± 0.08	0.66 ± 0.21	0.77 ± 0.14	0.73 ± 0.08	0.71 ± 0.06	0.71 ± 0.10	0.10 ± 0.12	0.71 ± 0.06	0.72 ± 0.08	0.66 ± 0.11	0.75 ± 0.13	0.72 ± 0.08
ICU <65 vs No_ICU <65	0.72 ± 0.08	0.69 ± 0.10	0.76 ± 0.13	0.73 ± 0.08	0.69 ± 0.08	0.71 ± 0.10	0.68 ± 0.09	0.69 ± 0.08	0.70 ± 0.09	0.70 ± 0.13	0.70 ± 0.09	0.71 ± 0.09
post_ICU vs control	0.95 ± 0.05	0.92 ± 0.10	0.95 ± 0.08	0.94 ± 0.06	0.84 ± 0.06	0.76 ± 0.18	0.81 ± 0.16	0.82 ± 0.08	0.93 ± 0.05	0.88 ± 0.15	0.94 ± 0.09	0.92 ± 0.07
ICU & No_ICU vs post_ICU & control	0.83 ± 0.04	0.91 ± 0.06	0.86 ± 0.05	0.79 ± 0.06	0.86 ± 0.04	0.92 ± 0.04	0.88 ± 0.04	0.82 ± 0.05	0.86 ± 0.04	0.91 ± 0.06	0.89 ± 0.04	0.83 ± 0.04
ICU vs No_ICU & post_ICU & control	0.72 ± 0.06	0.53 ± 0.11	0.63 ± 0.13	0.68 ± 0.05	0.77 ± 0.06	0.66 ± 0.13	0.69 ± 0.11	0.75 ± 0.06	0.76 ± 0.05	0.63 ± 0.10	0.63 ± 0.10	0.73 ± 0.06
ICU ≥65 vs ICU <65	0.54 ± 0.10	0.40 ± 0.19	0.46 ± 0.20	0.53 ± 0.10	0.53 ± 0.13	0.52 ± 0.18	0.48 ± 0.19	0.53 ± 0.13	0.60 ± 0.08	0.55 ± 0.17	0.56 ± 0.16	0.60 ± 0.09
ICU vs post_ICU	0.71 ± 0.06	0.83 ± 0.10	0.80 ± 0.08	0.60 ± 0.10	0.77 ± 0.09	0.90 ± 0.09	0.80 ± 0.08	0.64 ± 0.11	0.73 ± 0.09	0.81 ± 0.11	0.81 ± 0.10	0.65 ± 0.09
ICU vs No_ICU & control	0.79 ± 0.05	0.71 ± 0.14	0.70 ± 0.08	0.77 ± 0.07	0.80 ± 0.04	0.74 ± 0.11	0.71 ± 0.09	0.78 ± 0.05	0.77 ± 0.06	0.69 ± 0.14	0.71 ± 0.11	0.76 ± 0.07
ICU & pOST_ICU vs NO_ICU & control	0.75 ± 0.10	0.75 ± 0.08	0.5 ± 0.10	0.77 ± 0.05	0.78 ± 0.05	0.76 ± 0.06	0.76 ± 0.09	0.78 ± 0.05	0.76 ± 0.05	0.73 ± 0.08	0.73 ± 0.07	0.76 ± 0.05

### Shapley Additive Explanations Results

SHAP values can be used either to explain an instance behavior (the influence of the features in its output) or summarize to provide a global explanation of the model (e.g., averaging for all samples the SHAP-value of a feature to show its overall importance). The sign of a feature’s Shapley value provides information about the direction of its effect on a predictive model’s output. A positive Shapley value (SHAP-value) indicates that feature i contributes to the positive class of the predictive model for observation *x*. Conversely, a negative Shapley value indicates that feature *i* contributes to the negative class output. The Shapley value’s (SHAP-value) magnitude indicates how strongly the corresponding feature influences the model’s output. In our scenario, the SHAP-value Φ*k*,*i*(*x*) reports the contribution of the *it* *h* peak of the class. This contribution is an n-dimensional vector (n is the number of features or *m*/*z* values) that contains the average for all samples *x*. This vector shows the predictive importance with respect to a given classification task (e.g., COVID-19 vs control), and the highest absolute values are promising biomarker candidates. In our workflow for biomarker candidate discovery, we use KernelExplainer implementation,^28^ for SVM and MLP, and TreeExplainer implementation,^29^ XGBOOST. Figures 4 and 5 show the average and per data point Shapley values for the 20 features with the highest average contribution for “COVID-19 vs. Control”, “ICU vs Control”, “ICU vs Non_ICU”, and “Post_ICU vs Control” experiments, which have AUROC values with significant discriminatory power. Given that the tails of the distribution plots for each feature are colored with either the highest (red color) or lowest (blue color) feature value, we see that the prediction model is using either the presence or absence of peaks for a positive response (COVID-19, ICU, or Post-ICU).

**Figure 4:**
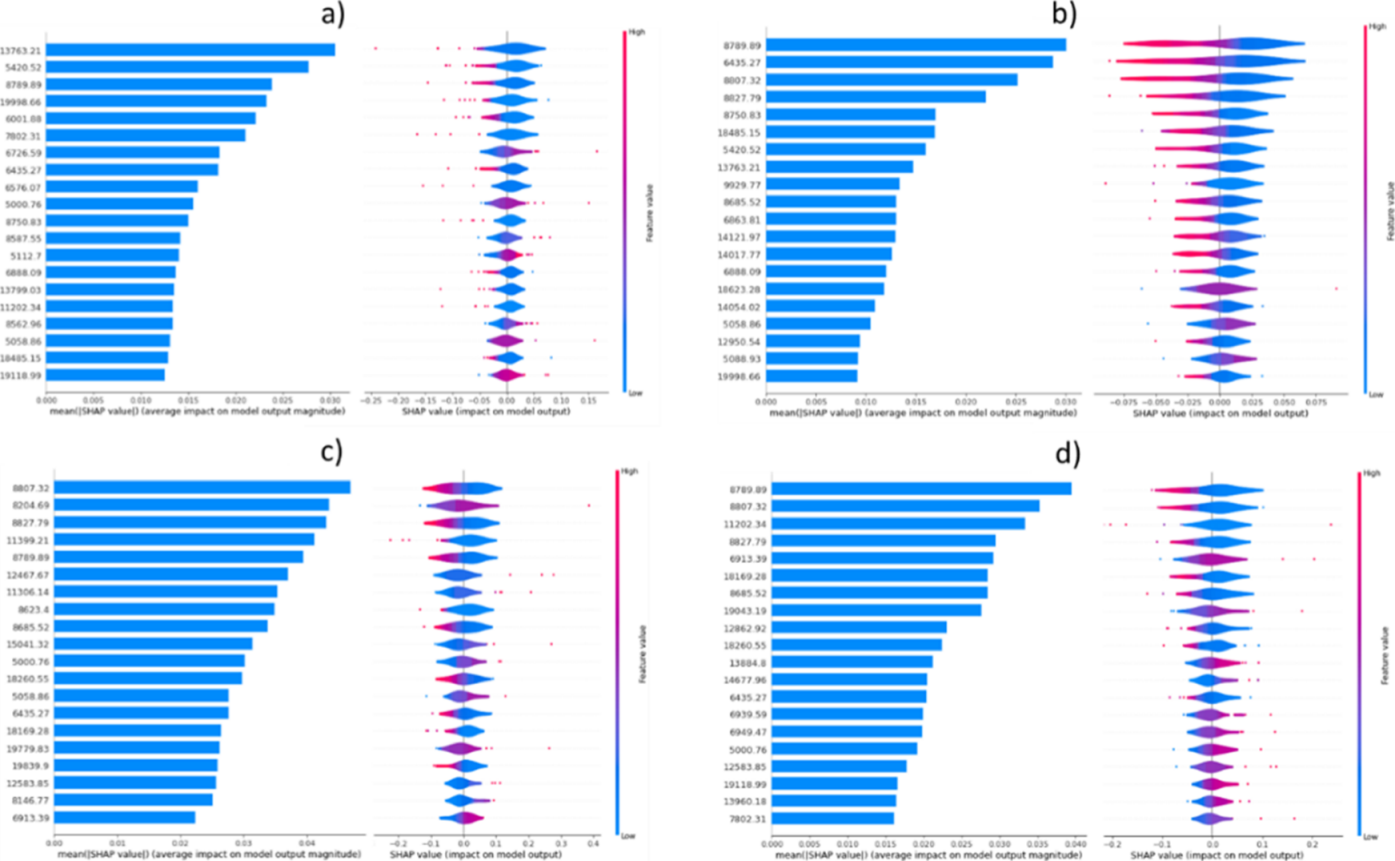
Quantification of feature impact on prediction analysis of Shapley additive explanations (SHAP) values of the 20 most impactful features a–d, for each of the most impactful experiments “COVID-19 vs. Control” (a), “ICU vs. Control” (b), “ICU vs. Non_ICU” (c), and “Post_ICU vs. Control” (d). The bar plot on the left indicates the average feature Shapley value that is related with the impact of the feature on the model output over all test samples. The colors of each spectrum at the right side indicate the feature value (low feature values are in blue and high one in red), and the positive or negative sides indicate if that feature has a positive influence or negative influence (SHAP value).

**Figure 5:**
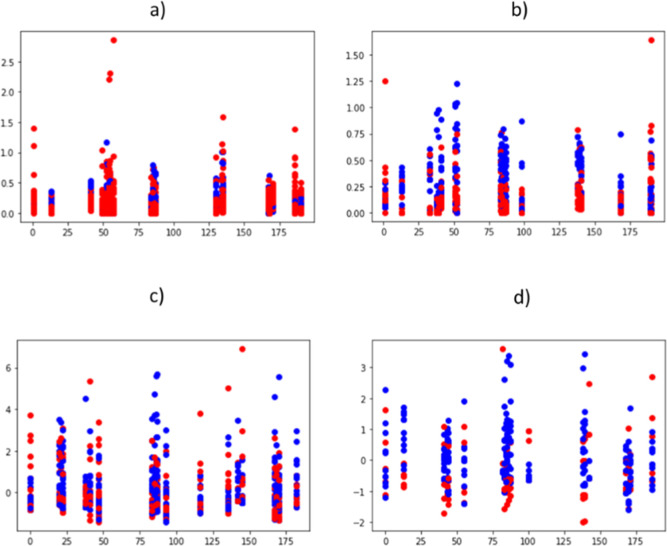
Visualization of original *m*/*z* 20 most impactful feature Shapley additive explanations (SHAP), for each of the most impactful experiments “COVID-19 vs. Control” (a), “ICU vs. Control” (b), “ICU vs. Non_ICU” (c), and “Post_ICU vs. Control” (d). The colors indicate the class that belongs to (blue is control and Non_ICU and red is COVID-19, ICU, and Post_ICU).

Among the top contributing features, *m*/*z* values 13,763.21, 5420.52, and 8789.89 are the most relevant in the model of COVID-19 vs control, whereas 8789.89, 6435.27, 8807.32, and 8827.79 are features that have a high impact in the ICU vs control model. The *m*/*z* 13,763 feature matched with serum prealbumin, also called transthyretin, lower concentrations of which in serum were associated with severe cases of COVID-19.^30^ The 8807.32 and 8827.79 features also have a high impact in the output ICU vs non- ICU model for predicting the severity of the course of the disease together with 8204.69 and 11,399.21. The fact that 8789.89, 8204.69, and 8827.79 are the most impactful features in the Post-ICU vs control model confirms their relevance. In different proteomics studies,^17,20^ the features with *m*/*z* ranging from 8700 to 8910 were associated with apolipoproteins C, APOC2 and APOC3, which are down-regulated in the serum of severe COVID-19 patients,^19^ suggesting a hyperinflammatory response and damaged vascular permeability.^31^ In fact, these features are possible biomarkers for patients being candidates for treatment in ICUs. The 8204.69 feature may correspond to complement factor 1, which is reported to have the function of humoral immune response,^32^ and 11,399.21 to serum amyloid A2 protein (SAA2), which were both reported in the cited proteomic studies. Moreover, SAA1 and A2 proteins were differentially expressed in high- and low-risk COVID-19 patients.^21^ Other proteins that were reported to be dysregulated in the sera of severe COVID-19 patients with adverse prognosis are Apolipoproteins C1, C2, and C3 and transthyretin.^21^ These results demonstrate the rationality of the featured SHAP peaks in the models. All of the identified proteins are found to be differentially expressed in COVID-19 patients and controls. These biomolecules are involved in SARS-CoV-2-related biological processes, such as amyloid fiber formation, platelet degranulation, humoral immune response, and inflammatory response. Platelet activation markers including PPBP (13,884) and platelet factor 4 PF4 (7800) were not identified as significant SHAP values in our calculations, but they contributed to the outputs of the COVID-19 vs control and Post-ICU vs control models.

Other contributing features could not be matched or identified with features reported in the literature for similar serum samples analyzed by MALDI-TOF-MS. SHAP feature maps provide insight into the specificity of the *m*/*z* feature biomarker candidates. The Shapley additive findings (SHAP) of the models can be further investigated by focusing on the top *m*/*z* features determined and performing a subsequent analysis using tandem MS on the most interesting selected *m*/*z* candidate ions to gather structural information.

Due to the fact that SHAP values had the same dimensions as the original mass spectra data sets, the principal component analysis (PCA) was used on the SHAP matrices from the data to evaluate the model predictions in low-dimensional space by grouping samples with similar features (Figure 6). The first three principal components (PCs) show that by selecting the top features based on their mean absolute SHAP values, the plot of the PCA scores is consistent with the model. Samples are mainly grouped by their class (category), indicating that feature contributions represented by SHAP values are group-specific. The divergence in the samples grouped to the ICU may be due to the impact of comorbidities (these results can be compared with the PCA clusters obtained using the raw *m*/*z* data (see Supporting Information); the improvement obtained using SHAP explanations can be seen visually).

**Figure 6:**
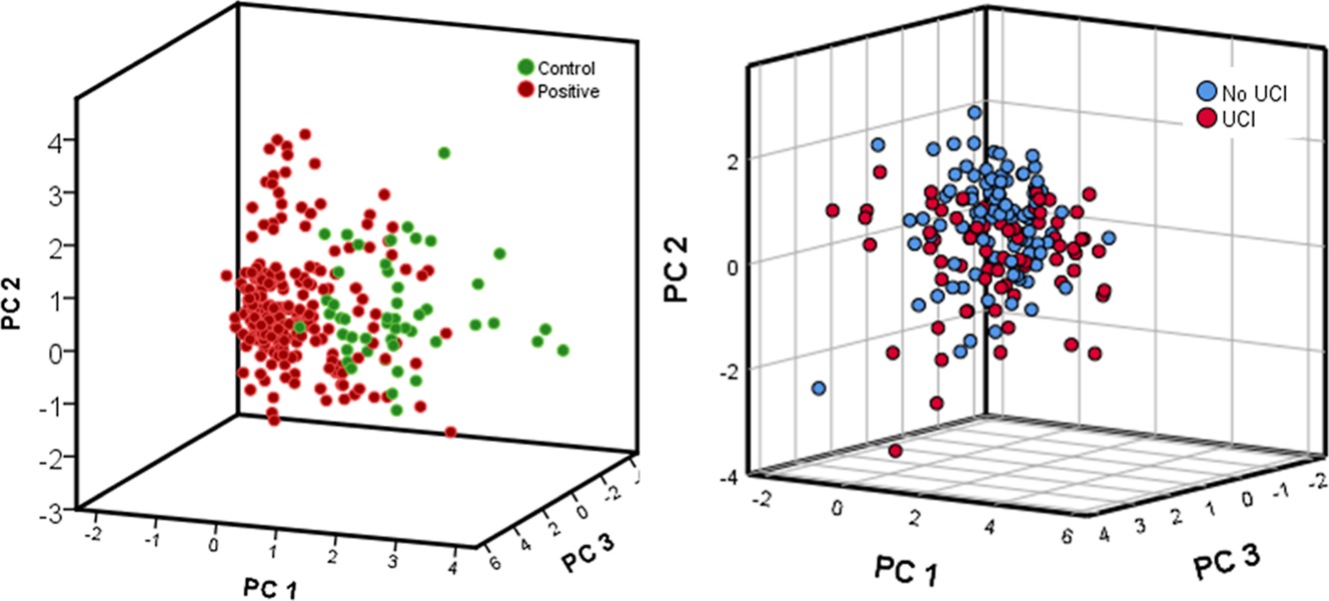
PCA performed on the selected variables according to SHAP values. Score plot for Control and COVID-19 groups on the left, and score plot for admission to ICU and not being admitted to ICU on the right.

## Conclusions

This study developed a MALDI-TOF MS method for the profiling of plasma samples. The machine-learning algorithm-trained model presented here is found to have an outstanding power of discrimination between controls and COVID-19 diagnosed patients, controls and patients in the ICU, and controls and patients who had been in the ICU. These results, therefore, suggest that these different groups must each have their corresponding spectral signatures. Moreover, there is a significant power of discrimination between ICU and Non_ICU patients. This is reflected in the significant differences in the spectral signatures between COVID-19 patients who were in the ICU and those who were never admitted to the ICU or had been discharged from the ICU. Hence, this model provides an efficient tool to separate patients infected with the SARS-CoV-2 virus by the severity of their symptoms and to indicate their prognosis in hospital settings. Among the top contributing features, we found a set of possible biomarkers that need to be further explored. Moreover, some of these have also been found in proteomic studies, demonstrating the rationality of the SHAP values. This combination of MALDI-TOF and ML represents an innovative approach for diagnosis and prognosis based on the analysis of the fingerprint data of serum samples. It is a simple, fast, cheap, and robust method that only requires minimal sample preparation and does not depend on the identification of mass spectra peaks through complex laboratory procedures.

## Methods

### Chemicals

Sinapinic acid (SA) was used as a matrix for MALDI-TOF MS analysis and was purchased from Bruker Daltonics (Bremen, Germany; #8201345). Trifluoroacetic acid (TFA) was purchased from Scharlab (#AC31420100; peptide synthesis grade), and acetonitrile (ACN) (mass spectrometry grade) was purchased from VWR (#83640.29). Protein Calibration Standard I (#8206355) was used for MALDI- TOF MS calibration and was purchased from Bruker Dal- tonics (Bremen, Germany). Ethylenediaminetetraacetic acid (EDTA) and 2-propanol were purchased from Merck KGaA, (Darmstadt, Germany). Ultrapure water was obtained by purification using a Milli-Q Plus system (Millipore Iberica S.A., Barcelona, Spain).

### Sample Collection

Samples and data from patients included in this study were provided by the Biobanks IDIBGI Biobank (B.0000872) and HUB-ICOIDIBELL (PT17/0015/0024), integrated in the Spanish National Biobanks Network and they were processed following standard operating procedures with the appropriate approval of the Ethics and Scientific Committees (Clinical Research Ethics Committee of the Doctor Josep Trueta Hospital in Girona ref#PEDIEC and IDIBELL ref#2020.088). A set of samples were collected during hospitalization from COVID-19 patients who had previously tested positive for severe acute respiratory syndrome coronavirus (SARS-CoV-2). Another set corresponded to control plasma samples collected during the year 2019 before the COVID-19 outbreak (pandemic). All samples were of peripheral blood stabilized in EDTA, centrifuged at 2000 g for 15 min at 20 °C, and stored at −80 °C. The characteristics of the samples used in this study are Control SARS-CoV-2-negative (*n* = 50, age not declared); SARS-CoV-2-positive patients age 65: none staying at ICU (*n* = 50), staying at ICU (*n* = 50), post ICU (22); SARS-CoV-2-positive patients age ≤65: none staying at ICU (*n* = 52), admitted at ICU (*n* = 40), post ICU (*n* = 11). Since we were unable to access the patients’ medical records, we are unable to determine whether the patients also had additional disorders. The patients’ samples were processed in the molecular area of the territorial laboratory of Girona under certified class II biological safety laboratory and kept, wearing all the appropriate personal protective equipment required by the World Health Organization to perform PCR diagnostics. The RT-PCR diagnosis was performed using two different methodological platforms which detect two targets (N and E qRT-PCR methodology, Xpert SARS-CoV-2, Cepheid, US) or four targets (N, E, S and RpRd genes, Allplex 2019-nCoV assay, Seegen, South Korea).

### Sample Preparation for MALDI-TOF MS

Samples of plasma were thawed and processed inside a Biosafety II Cabin (cf. Sample Collection section). All samples were first diluted ten times with a 0.1% TFA solution in Milli-Q water. They were then mixed in a 1:1 ratio with a solution of SA containing 20 mg of SA/mL in 60%:40% (v/v) ACN:Milli-Q water. Finally, 1 μL of the mixture was spotted on a purified stainless-steel target plate (MTP 384 target ground steel; Bruker Daltonics, Bremen, Germany) in triplicate and allowed to dry at room temperature before being analyzed by MALDI-TOF MS. In order to prevent the risks of carry-over contamination, the target plate was regularly cleaned in an ultrasonic bath using a specific cleaning procedure with ultrapure solvents, applying 2-propanol, Milli-Q water, 2-propanol, and TA30 (350 mL ACN: 350 mL TFA 0.1%) sequentially.

### Acquisition of Mass Spectra Using Matrix-Assisted Laser Desorption Ionization (MALDI TOF MS)

Mass spectra were acquired using Autoflex maX with Time-Of-Flight (TOF) analyzer from Bruker Daltonics (Bruker Daltonics, Bremen, Germany). Ionization was achieved by irradiation with a solid phase laser (with patented Smart beam technology) operating at 2000 Hz. All spectra were automatically acquired using a regular raster (in random walk mode), and 20 shots were randomly made in each raster spot; locations were calibrated prior to each run. Sample mass spectra correspond to the sum of 1800 shots taken in 300-shot cycles. All measurements were carried out in the positive linear mode, and each spectrum was externally calibrated using a standard mixture of peptides (Protein Calibration Standard I). All mass spectra were acquired using FlexControl software (Bruker Daltonics, Germany) and each spectrum consisted of more than 25,000 mass-to-charge (*m*/*z*) values with the corresponding intensities in the 5 to 20 kDa mass range. The smoothing of mass spectra by the Savitzky–Golay method, the baseline subtraction by the Top-Hat method, and the recalibration of each mass spectrum, followed by export as an ASCII file, was performed using the FlexAnalysis 3.4 software (Bruker Daltonics, Germany), thus obtaining a 191-dimensional vector representation for each sample.

### Statistical Analysis

The data for patients and healthy controls were divided into test and validation data sets for statistical analysis. All acquired mass spectra were baseline subtracted. Intensities of peaks at defined mass-to-charge (*m*/*z*) values that reproducibly exceeded the signal-to-noise ratio of three were included in analyses. Box and Whiskers plots and statistical analyses including factor analysis (eigenvalues), PC analyses (PCA), and linear discrimination analysis were performed with SPSS 25.0 for Windows.

### Machine Learning

In order to classify the SARS-CoV-2 positive and negative samples, a ML approach was used. The learned model represents the best solution given the data samples obtained by MALDI-TOF MS from different experiments. To study the performance, three of the most well-known and successfully widely applied techniques–deep neural network classifier (multilayer perceptron, MLP), eXtreme Gradient BOOSTing Trees (XGBOOST),^33^ and SVMs^34^–were selected and their parameters were adjusted to obtain their best results to the problem under consideration. A cross-validation (CV) was applied to study the performance of the three techniques. A number of *K* = 10-folders was used since the number of samples was small, but a minimum of four positive and negative examples were added as a constraint for each fold to ensure that there were samples of at least two different individuals. It should be noted that 20% of the samples were randomly separated before the CV process in order to perform the test using the best model selected. The CV test was performed 20 times to avoid bias and guarantee that the results were independent of the samples selected in the training phase (double-blind test). We report the area under the receiver operating characteristics curve (AUROC), positive sensitivity, positive specificity, and accuracy as performance metrics. The data sets of most of the experiments have a high-class imbalance (50 controls, 100 Non_ICU, 33 post-ICU, and 92 ICU). The invariant capabilities of the AUROC metric with respect to the class ratio of the data set allow a fair comparison between different individuals’ COVID-19 state class ratios, but they do not reflect the sensitivity or specificity of the positive class. For this reason, we have included the sensitivity and specificity of the best model for each experiment in the results. Hence, we set AUROC as the objective optimization metric for adjusting ML hyperparameters, and the positive sensitivity, positive specificity, and accuracy are reported as performance metrics. It is worth remembering that AUROC shows the true-positive rate versus the false-positive rate and can be understood as the probability of correctly classifying a pair of samples. A random classifier would obtain 0.5 for AUROC, and values above 0.7 are considered acceptable for discrimination between groups. We can consider a set of input/output pairs *S* = {(*x*_1_, *y*_1_), (*x*_2_, *y*_2_), ..., (*x*_*n*_, *y*_*n*_)}, where *X* inputs belongs to R ^*d*^ and the *Y* outputs to {0, 1}, being *n* the number of samples. We set 0 as the standard model and 1 as the hypothesis with which we want to compare it with. The question to be answered is whether the hypothesis follows the standard model or if there is a different distribution (in our case, a difference in distribution relates to different spectral signatures). The ML approach estimates of *p*(*y* |*X*) being *Y* = {0, 1} by obtaining an empirical model solution that minimizes the empirical error $\frac{1}{n}\Sigma_{i=1}^{n}{\left(y_{i}-f\left(x_{i}\right)\right)}^{2}$ ^35^ and allows the formulation of a preliminary hypothesis as binary questions (e.g., is there any difference between detecting SARS-CoV-2 for people under 65 and over 65 years old?). Thus, the model’s ability to learn the classification problem reveals the existence of a signature that allows discrimination between groups. In this case, a posterior explainability analysis is performed to obtain information about the pattern of the signature. We designed a set of experiments to elucidate the extent to which the combination of MALDI-TOF analyses and ML may be able to provide a useful diagnostic and prognostic tool for clinical use and whether age is an important factor in the prognosis. We used AUROC as a performance metric to optimize during the model selection process. Moreover, we also provided performance metrics F1, sensitivity, and specificity

$$F1-score=\frac{2\cdot specificity\cdot sensitivity}{specificity+sensitivity}$$

$$Specificity=\frac{TP}{TP+FP}$$

$$Sensitivity=\frac{TP}{P}$$

### Analysis of Feature Contribution through Shapley Additive exPlanations (SHAP)

Single peaks are generally used rather than full mass spectrum information in prediction applications. Digital biomarkers that are generated by using ML do not need to be viewed as a black box. Instead, explainable AI techniques are available that can provide more granular details about the explanatory variables that influence the prediction made. In our case, we wanted to assess whether the predictive performance of our models is primarily driven by a single subset of the peaks or whether the full spectrum is used. Shapley Additive exPlanations (SHAP)^36^ calculates the mean marginal contribution to the output of the model of each feature in each instance. It is described as a model-agnostic interpretability method as it can derive posthoc explanations for the predictions of any type of ML model by analysis of the changes of the outputs associated with slightly perturbed inputs. We used and adapted original SHAP formula for a multilabeled classification setting as defined in eq 1

$$\phi_{k,i}\left(x\right)=\sum_{S\in F\left\{i\right\}}\frac{\left|S\right|!\left(\left|F\right|-\left|S\right|-1\right)!}{\left|F\right|!}\left[f_{k,S\cup \left\{i\right\}}\left(x_{S\cup \left\{i\right\}}\right)-f_{k,S}\left(x_{S}\right)\right]$$

*F* being all the feature of the data set and *f*_*k*,*S*_ () a function, built upon a partition *S* of the set of features, that returns 1 if for the data point *x*_*s*_, the label *k* provided by the model is *k* (note that this particular notation makes it explicit that we are provided with a set of explanations, one for each feature, that are label-specific). The workflow for biomarker candidate discovery is listed in Figure 7.

**Figure 7:**
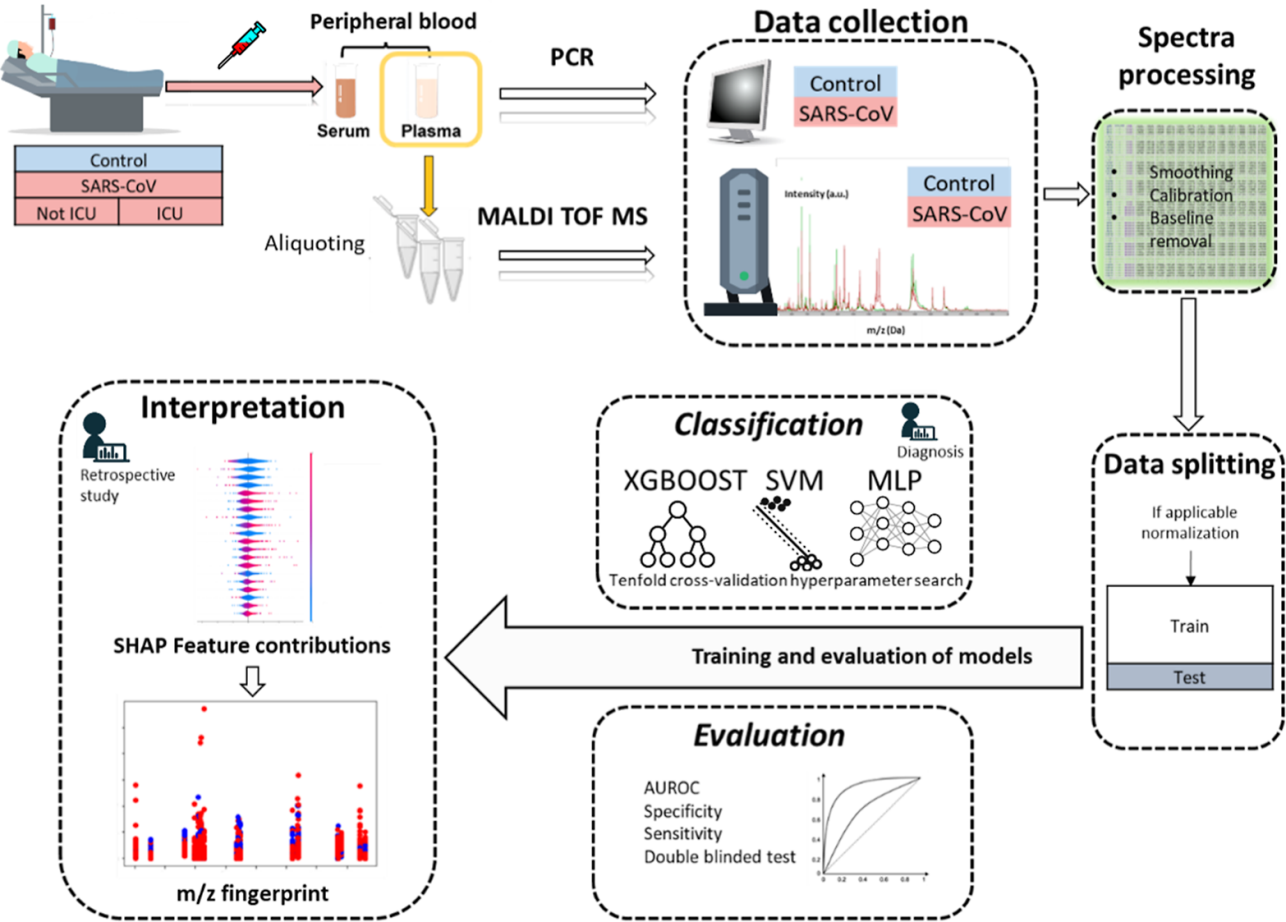
Workflow for biomarker candidates for MALDI-TOF-MS-based serum detection of SARS-CoV-2.

## Supplementary Material



## Notes

Information about PC and discriminant analysis studies; three-dimensional score plot from PCA showing the distinction trends between control and SARS-CoV-2 samples using data from *m*/*z* regions 11,000–12,000 (A) and 13,800–14,800 (B); (A) example of mass spectra for control and SARS-CoV-2 positive samples; PCA score plots of an unsupervised PCA model displaying PCs (PC) 1, 2, and 3 using the mass range *m*/*z* 5–20 kDa with the mass spectra of the plasma for (B) control (green) and COVID-19 (red) groups (C) control, ICU, Post-ICU, and Non-ICU categories, and (D) age; coloring according to group; discriminant analysis performed on the selected variables according to top SHAP values in Figure 5 (c) considering control, admitted to ICU and not admitted to ICU; and discriminant analysis results considering A: all *m*/*z* values and B: Shapley variables selected (PDF)

## #

HIPRA, Avinguda La Selva, 135, 17170 Amer, Girona, Catalonia, Spain

## References

[1] Lazari L. C., Zerbinati R. M., Rosa-Fernandes L., Santiago V. F., Rosa K. F., Angeli C. B., Schwab G., Palmieri M., Sarmento D. J. S., Marinho C. R. F., Almeida J. D., To K., Giannecchini S., Wrenger C., Sabino E. C., Martinho H., Lindoso J. A. L., Durigon E. L., Braz-Silva P. H., Palmisano G. (2022). MALDI-TOF Mass Spectrometry of Saliva Samples as a
Prognostic Tool for COVID-19. J. Oral Microbiol.

[2] Sivanesan I., Gopal J., Surya Vinay R., Luke E. H., Oh J. W., Muthu M. (2022). Consolidating the Potency
of Matrix-Assisted Laser Desorption/Ionization-Time
of Flight Mass Spectrometry (MALDI-TOF MS) in Viral Diagnosis: Extrapolating
Its Applicability for COVID Diagnosis?. TrAC,
Trends Anal. Chem.

[3] Filchakova O., Dossym D., Ilyas A., Kuanysheva T., Abdizhamil A., Bukasov R. (2022). Review of COVID-19
Testing and Diagnostic
Methods. Talanta.

[4] Cobo F. (2013). Application
of MALDI-TOF Mass Spectrometry in Clinical Virology: A Review. Open Virol J..

[5] Patel R. (2015). MALDI-TOF
MS for the Diagnosis of Infectious Diseases. Clin Chem..

[6] Teunissen C. E., Koel-Simmelink M., Pham T. V., Knol J. C., Khalil M., Trentini A., Killestein J., Nielsen J., Vrenken H., Popescu V., Dijkstra C. D., Jimenez C. R. (2011). Identification of
Biomarkers for Diagnosis and Progression of MS by MALDI-TOF Mass Spectrometry. Mult. Scler. J.

[7] Iles R. K., Zmuidinaite R., Iles J. K., Carnell G., Sampson A., Heeney J. L. (2020). Development of a Clinical MALDI-ToF
Mass Spectrometry
Assay for SARS-CoV-2: Rational Design and Multi-Disciplinary Team
Work. Diagnost.

[8] Seethi V. D. R., LaCasse Z., Chivte P., Bland J., Kadkol S. S., Gaillard E. R., Bharti P., Alhoori H. (2024). An Explainable
AI Approach
for Diagnosis of COVID-19 Using MALDI-ToF Mass Spectrometry. Expert Syst. Appl..

[9] Deulofeu M., Kolářová L., Salvadó V., María Peña-Méndez E., Almaši M., Štork M., Pour L., Boadas-Vaello P., Ševčíková S., Havel J., Vaňhara P. (2019). Rapid Discrimination
of Multiple Myeloma Patients by Artificial Neural Networks Coupled
with Mass Spectrometry of Peripheral Blood Plasma. Sci. Rep.

[10] Nachtigall F. M., Pereira A., Trofymchuk O. S., Santos L. S. (2020). Detection of SARS-CoV-2
in Nasal Swabs Using MALDI-MS. Nat. Biotechnol..

[11] Deulofeu M., García-Cuesta E., Peña-Méndez E. M., Conde J. E., Jiménez-Romero O., Verdú E., Serrando M. T., Salvadó V., Boadas-Vaello P. (2021). Detection
of SARS-CoV-2 Infection in Human Nasopharyngeal Samples by Combining
MALDI-TOF MS and Artificial Intelligence. Front
Med..

[12] Chivte P., LaCasse Z., Seethi V. D. R., Bharti P., Bland J., Kadkol S. S., Gaillard E. R. (2021). MALDI-ToF Protein
Profiling as a
Potential Rapid Diagnostic Platform for COVID-19. J. Mass Spectrom. Adv. Clin. Lab.

[13] Rocca M. F., Zintgraff J. C., Dattero M. E., Santos L. S., Ledesma M., Vay C., Prieto M., Benedetti E., Avaro M., Russo M., Nachtigall F. M., Baumeister E. (2020). A Combined Approach of MALDI-TOF
Mass Spectrometry and Multivariate Analysis as a Potential Tool for
the Detection of SARS-CoV-2 Virus in Nasopharyngeal Swabs. J. Virol. Methods.

[14] Shen B., Yi X., Sun Y., Bi X., Du J., Zhang C., Quan S., Zhang F., Sun R., Qian L., Ge W., Liu W., Liang S., Chen H., Zhang Y., Li J., Xu J., He Z., Chen B., Wang J., Yan H., Zheng Y., Wang D., Zhu J., Kong Z., Kang Z., Liang X., Ding X., Ruan G., Xiang N., Cai X., Gao H., Li L., Li S., Xiao Q., Lu T., Zhu Y., Liu H., Chen H., Guo T. (2020). Proteomic
and Metabolomic Characterization
of COVID-19 Patient Sera. Cell.

[15] Park J., Kim H., Kim S. Y., Kim Y., Lee J. S., Dan K., Seong M. W., Han D. (2020). In-Depth Blood
Proteome Profiling
Analysis Revealed Distinct Functional Characteristics of Plasma Proteins
between Severe and Non-Severe COVID-19 Patients. Sci. Rep.

[16] Whetton A. D., Preston G. W., Abubeker S., Geifman N. (2020). Proteomics and Informatics
for Understanding Phases and Identifying Biomarkers in COVID-19 Disease. J. Proteome Res..

[17] Nuñez E., Orera I., Carmona-Rodríguez L., Paño J. R., Vázquez J., Corrales F. J. (2022). Mapping the Serum
Proteome of COVID-19
Patients; Guidance for Severity Assessment. Biomedicines.

[18] Kimura Y., Nakai Y., Shin J., Hara M., Takeda Y., Kubo S., Jeremiah S. S., Ino Y., Akiyama T., Moriyama K., Sakai K., Saji R., Nishii M., Kitamura H., Murohashi K., Yamamoto K., Kaneko T., Takeuchi I., Hagiwara E., Ogura T., Hasegawa H., Tamura T., Yamanaka T., Ryo A. (2021). Identification of Serum
Prognostic Biomarkers of Severe COVID-19 Using a Quantitative Proteomic
Approach. Sci. Rep..

[19] Delafiori J., Navarro L. C., Siciliano R. F., De Melo G. C., Busanello E. N. B., Nicolau J. C., Sales G. M., De Oliveira A. N., Val F. F. A., De Oliveira D. N., Eguti A., Dos Santos L. A., Dalçóquio T. F., Bertolin A. J., Abreu-Netto R. L., Salsoso R., Baía-da-Silva D., Marcondes-Braga F. G., Sampaio V. S., Judice C. C., Costa F. T. M., Durán N., Perroud M. W., Sabino E. C., Lacerda M. V. G., Reis L. O., Fávaro W. J., Monteiro W. M., Rocha A. R., Catharino R. R. (2021). Covid-19
Automated Diagnosis and Risk Assessment through Metabolomics and Machine
Learning. Anal. Chem..

[20] Yan L., Yi J., Huang C., Zhang J., Fu S., Li Z., Lyu Q., Xu Y., Wang K., Yang H., Ma Q., Cui X., Qiao L., Sun W., Liao P. (2021). Rapid Detection of
COVID-19 Using MALDI-TOF-Based Serum Peptidome Profiling. Anal. Chem..

[21] Lazari L. C., Ghilardi F. D. R., Rosa-Fernandes L., Assis D. M., Nicolau J. C., Santiago V. F., Dalçóquio T. F., Angeli C. B., Bertolin A. J., Marinho C. R. F., Wrenger C., Durigon E. L., Siciliano R. F., Palmisano G. (2021). Prognostic
Accuracy of MALDI-TOF
Mass Spectrometric Analysis of Plasma in COVID-19. Life Sci. Alliance.

[22] Gomila R. M., Martorell G., Fraile-Ribot P. A., Doménech-Sánchez A., Albertí M., Oliver A., García-Gasalla M., Albertí S. (2021). Use of Matrix-Assisted Laser Desorption Ionization
Time-of-Flight Mass Spectrometry Analysis of Serum Peptidome to Classify
and Predict Coronavirus Disease 2019 Severity. Open Forum Infect Dis.

[23] Bourgin M., Durand S., Kroemer G. (2023). Diagnostic, Prognostic and Mechanistic
Biomarkers of COVID-19 Identified by Mass Spectrometric Metabolomics. Metabolites.

[24] Cardinal-Fernández P., Garcia Cuesta E., Barberán J., Varona J. F., Estirado A., Moreno A., Villanueva J., Villareal M., Baez-Pravia O., Menéndez J., Villares P., López
Escobar A., Rodríguez-Pascual J., Almirall C., Domínguez E., Pey C., Ferreiro A., Revilla
Amores M., Sánchez N., Ruiz de Aguiar S., Castellano J. M. (2021). Clinical Characteristics and Outcomes of 1,331 Patients
with Covid-19: Hm Spanish Cohort. Rev. Esp.
Quimioter.

[25] Yatim N., Boussier J., Chocron R., Hadjadj J., Philippe A., Gendron N., Barnabei L., Charbit B., Szwebel T. A., Carlier N., Pène F., Azoulay C., Khider L., Mirault T., Diehl J. L., Guerin C. L., Rieux-Laucat F., Duffy D., Kernéis S., Smadja D. M., Terrier B. (2021). Platelet Activation
in Critically Ill COVID-19 Patients. Ann. Intensive
Care.

[26] Hurler L., Szilágyi Á., Mescia F., Bergamaschi L., Mező B., Sinkovits G., Réti M., Müller V., Iványi Z., Gál J., Gopcsa L., Reményi P., Szathmáry B., Lakatos B., Szlávik J., Bobek I., Prohaszka Z. Z., Förhécz Z., Csuka D., Kajdácsi E., Cervenak L., Kiszel P., Masszi T., Vályi-Nagy I., Würzner R., Lyons P. A., Toonen E. J. M., Prohaszka Z. (2023). Complement
Lectin Pathway Activation Is Associated with COVID-19 Disease Severity,
Independent of MBL2 Genotype Subgroups. Front
Immunol.

[27] Demichev V., Tober-Lau P., Lemke O., Nazarenko T., Thibeault C., Whitwell H., Röhl A., Freiwald A., Szyrwiel L., Ludwig D., Correia-Melo C., Aulakh S. K., Helbig E. T., Stubbemann P., Lippert L. J., Grüning N. M., Blyuss O., Vernardis S., White M., Messner C. B., Joannidis M., Sonnweber T., Klein S. J., Pizzini A., Wohlfarter Y., Sahanic S., Hilbe R., Schaefer B., Wagner S., Mittermaier M., Machleidt F., Garcia C., Ruwwe-Glösenkamp C., Lingscheid T., Bosquillon de Jarcy L., Stegemann M. S., Pfeiffer M., Jürgens L., Denker S., Zickler D., Enghard P., Zelezniak A., Campbell A., Hayward C., Porteous D. J., Marioni R. E., Uhrig A., Müller-Redetzky H., Zoller H., Löffler-Ragg J., Keller M. A., Tancevski I., Timms J. F., Zaikin A., Hippenstiel S., Ramharter M., Witzenrath M., Suttorp N., Lilley K., Mülleder M., Sander L. E., Ralser M., Kurth F., Kleinschmidt M., Heim K. M., Millet B., Meyer-Arndt L., Hübner R. H., Andermann T., Doehn J. M., Opitz B., Sawitzki B., Grund D., Radünzel P., Schürmann M., Zoller T., Alius F., Knape P., Breitbart A., Li Y., Bremer F., Pergantis P., Schürmann D., Temmesfeld-Wollbrück B., Wendisch D., Brumhard S., Haenel S. S., Conrad C., Georg P., Eckardt K. U., Lehner L., Kruse J. M., Ferse C., Körner R., Spies C., Edel A., Weber-Carstens S., Krannich A., Zvorc S., Li L., Behrens U., Schmidt S., Rönnefarth M., Dang-Heine C., Röhle R., Lieker E., Kretzler L., Wirsching I., Wollboldt C., Wu Y., Schwanitz G., Hillus D., Kasper S., Olk N., Horn A., Briesemeister D., Treue D., Hummel M., Corman V. M., Drosten C., von Kalle C. (2021). A Time-Resolved Proteomic and Prognostic
Map of COVID-19. Cell Syst.

[28] shap .KernelExplainer—SHAP latest documentation. https://shap.readthedocs.io/en/latest/generated/shap.KernelExplainer.html (accessed Jul,19 2023).

[29] shap .TreeExplainer—SHAP latest documentation. https://shap.readthedocs.io/en/latest/generated/shap.TreeExplainer.html (accessed Jul,17 2025).

[30] Zinellu A., Mangoni A. A. (2021). Serum Prealbumin
Concentrations, COVID-19 Severity,
and Mortality: A Systematic Review and Meta-Analysis. Front Med. (Lausanne).

[31] Zhang Y., Cai X., Ge W., Wang D., Zhu G., Qian L., Xiang N., Yue L., Liang S., Zhang F., Wang J., Zhou K., Zheng Y., Lin M., Sun T., Lu R., Zhang C., Xu L., Sun Y., Zhou X., Yu J., Lyu M., Shen B., Zhu H., Xu J., Zhu Y., Guo T. (2022). Potential Use of Serum
Proteomics for Monitoring COVID-19 Progression to Complement RT-PCR
Detection. J. Proteome Res..

[32] Shu T., Ning W., Wu D., Xu J., Han Q., Huang M., Zou X., Yang Q., Yuan Y., Bie Y., Pan S., Mu J., Han Y., Yang X., Zhou H., Li R., Ren Y., Chen X., Yao S., Qiu Y., Zhang D. Y., Xue Y., Shang Y., Zhou X. (2020). Plasma Proteomics Identify Biomarkers
and Pathogenesis of COVID-19. Immunity.

[33] Chen, T. ; Guestrin, C. XGBoost: A Scalable Tree Boosting System. Proceedings of the 22nd ACM SIGKDD International Conference on Knowledge Discovery and Data Mining, 2016.

[34] Boser, B. E. ; Guyon, I. M. ; Vapnik, V. N. Training Algorithm for Optimal Margin Classifiers. In Proceedings of the Fifth Annual ACM Workshop on Computational Learning Theory, 1992; pp 144–152.

[35] Shalev-Shwartz, S. ; Ben-David, S. Understanding Machine Learning: From Theory to Algorithms; Cambridge University Press, 2013. 10.1017/CBO9781107298019.

[36] Lundberg, S. M. ; Allen, P. G. ; Lee, S.-I. A Unified Approach to Interpreting Model Predictions. NIPS’17: Proceedings of the 31st International Conference on Neural Information Processing Systems 2017, 30, 4768–4777

