# Supplementary material for: MALDI-TOF MS and Machine Learning Explanations for the Detection of SARS-CoV‑2 Infection in Human Plasma: Fingerprints as a Strategy for Risk Assessment
Source: ACS Omega. 2025 Sep 8;10(36):41413–24. doi: 10.1021/acsomega.5c04593 (PMC12444529; doi:10.1021/acsomega.5c04593)
Supplement: Supplementary file 1 [file ao5c04593_si_001.pdf]

Supporting Information for:

MALDI-TOF MS and Machine Learning Explanations for the Detection of SARS-CoV-2 Infection in Human Plasma: Fingerprints as a Strategy for Risk Assessment

Meritxell Deulofeu [†,a], *Esteban García-Cuesta* [‡,*], *Eladia María Peña-Méndez* [§], *José Elías Conde-González*[§], *Orlando Jiménez-Romero* [†,ǂ], *Enrique Verdú* [†], *Maria Teresa Serrando* [†,ǂ], *Victoria Salvadó* [§] *and Pere Boadas-Vaello* [†,*]

[†]Research Group of Clinical Anatomy, Embryology and Neuroscience (NEOMA), Department of Medical Sciences, University of Girona, Girona, Catalonia, Spain

[‡]Department of Artificial Intelligence, Universidad Politécnica de Madrid, Madrid, Spain.

[§]Department of Chemistry, Analytical Chemistry Division, Faculty of Sciences, University of La Laguna, 38204 San Cristóbal de La Laguna, Tenerife, Spain

[ǂ] ICS-IAS Girona Clinical Laboratory, Santa Caterina Hospital, Parc Sanitari Martí i Julià, Salt, Catalonia, Spain

[§]Department of Chemistry, Faculty of Science, University of Girona, 17071 Girona, Catalonia, Spain

[*]**Corresponding Authors**: Dr. Esteban García-Cuesta and Dr. Pere Boadas-Vaello. Address for correspondence: esteban.garcia@fi.upm.es and pere.boadas@udg.edu

[a] Present address: HIPRA, Avinguda La Selva, 135, 17170 Amer, Girona, Catalonia, Spain

KEYWORDS: COVID-19 risk assessment; MALDI-TOF MS; ML explicability; COVID-19 fingerprints

Principal Component study

The potential of MALDI-TOF serum profiling as a single-domain assay to simultaneously predict multifactor onset is evaluated. Principal component analysis (PCA) reduces high-dimensional data complexity while preserving trends and patterns, which is accomplished by reducing the number of dimensions in the data. PCA makes it possible to perform analyses based on fingerprints and permits easy visualization of the high dimensional data set. Examination of trends in samples was made by directly comparing the Control and SARS-CoV-2 (Positive) group, and within the SARS-CoV-2 (positive) group by studying age, not having been in the ICU, being in the ICU and having been in the ICU. Fist at all, PCA was carried out using all the m/z data of 5–20 kDa mass range (**Fig. S1 A**) corresponding to the different groups (Control, Staying in ICU, non-staying in ICU, and post-ICU). The results provide a means for grouping the Control and COVID-19 samples (**Fig. S1** B). Beyond the characteristic patterns for Control and COVID-19 diagnosed samples, the variance in age and factors such as having been in the ICU or not did not reveal clear differences (**Fig. S1** C). The PCA outputs displayed many differences between individual samples of particular groups. Furthermore, it should be considered that the increasing number of SARS- CoV-2 mutations is resulting in a greater variety of modified viruses with distinct signatures, all of which have their corresponding secondary health problems. If the mutations and samplings are random, the original virus will be near the data mean.

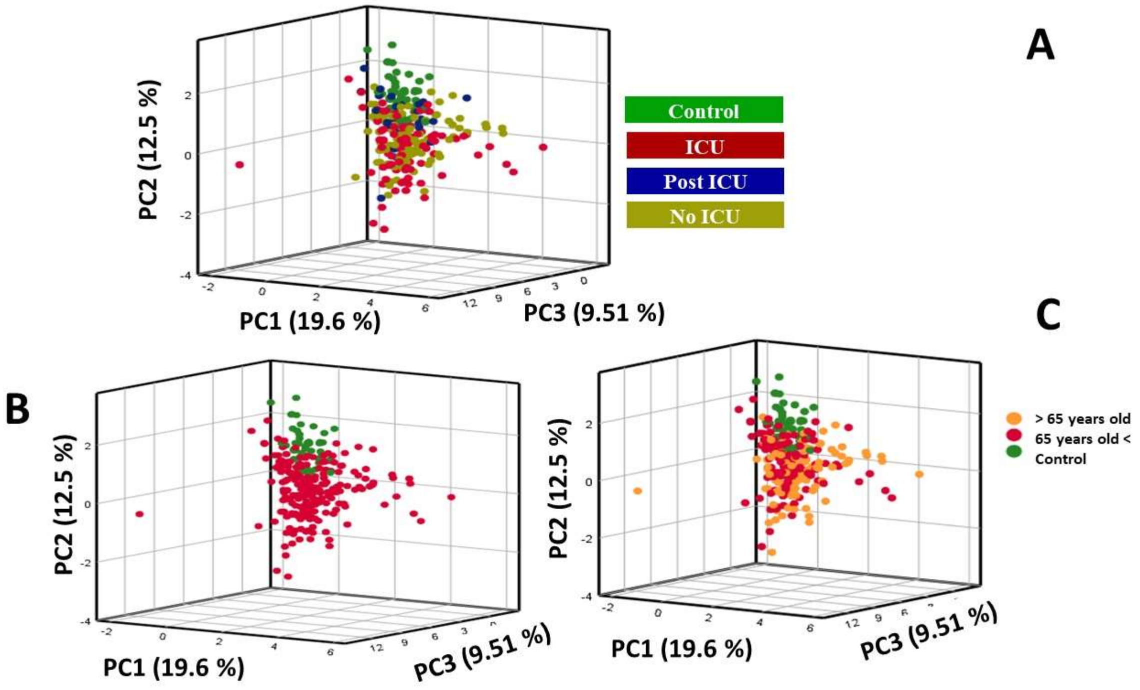

Figure S1: PCA score plots of an unsupervised PCA model displaying principal components (PC) 1, 2 and 3 using the mass range m/z 5–20 kDa with the mass spectra of the plasma for (A) Control, ICU, Post-ICU and Non-ICU categories, (B) Control (green) and SARS-CoV (red) groups, and (C) Age; colouring according to group.

Principal component analysis (PCA) was then applied to the mass spectra data obtained for the Control and Positive SARS-CoV-2 (positive) groups for m/z regions 11,000-12,000 and 13,800-14,800 (**Fig. S2**). Comparison of the trends observed in samples only revealed an improvement in the differentiation between Control and Positive samples when the m/z region 13,800- 14,800 was used. There was little variation by the subgroups within the Positive group.

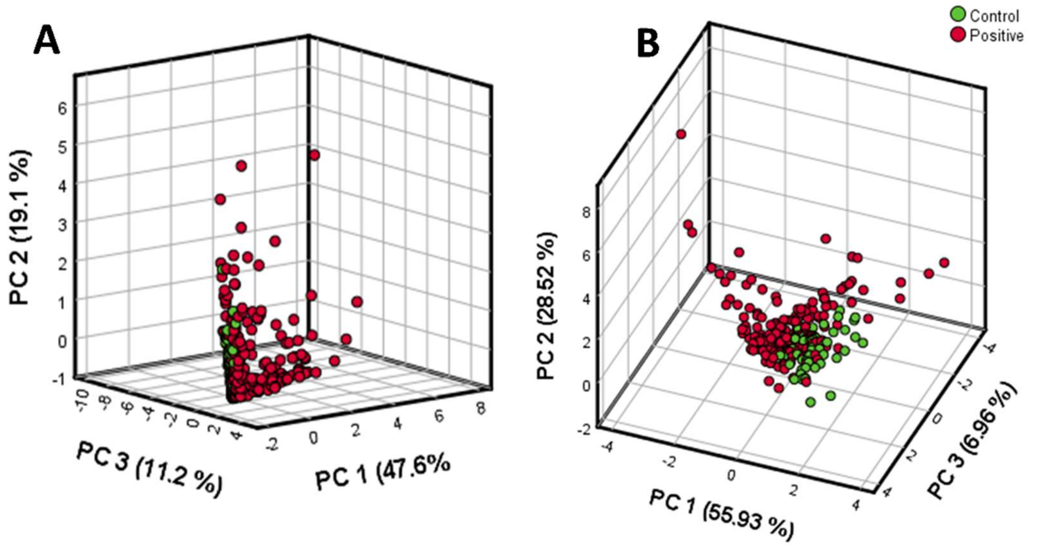

Figure S2. Three-dimensional score plot from PCA showing the distinction trends between Control and SARS-CoV-2 samples using data from m/z regions 11,000-12,000 (A) and 13,800- 14,800 (B).

Discriminant analysis study

A new group of samples consisting of Post-ICU and ICU was created after which a classification was made as follows: Control, Not having been admitted to the ICU and the new group. This classification was based on the Shapley variables (Fig. 5 (c)). The resulting discriminant analysis (DA) outperformed comparable models by reaching accuracies of 76.8% (73.5 % after cross validation), resulting in improved classification results (**Fig. S3**).

The differences on the transformed LDA space with and without SHAP feature variable selection is showed in figure S4.

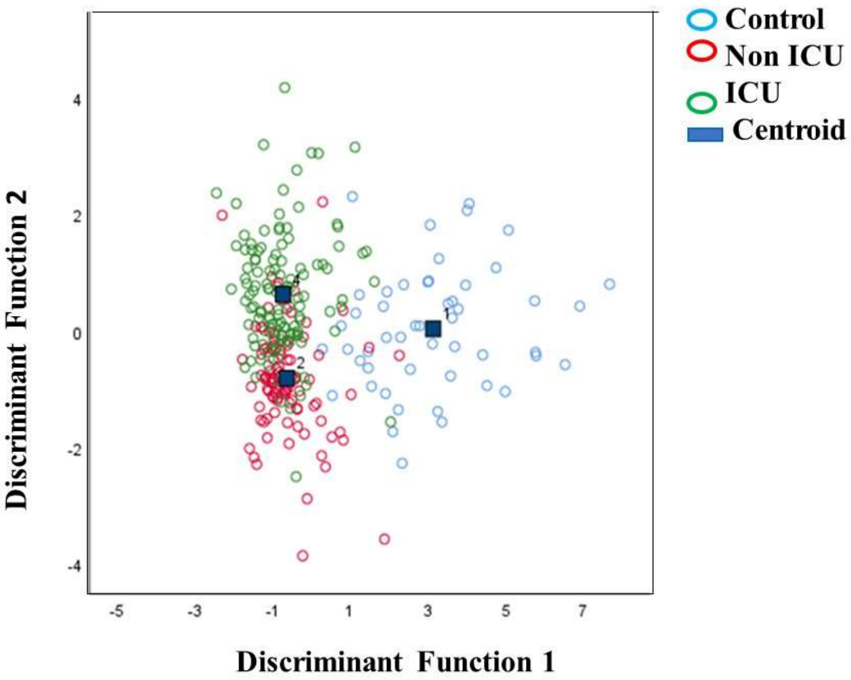

Confusing Table

Class	Predicted Class		
	Control	*Non ICU*	*ICU*
Control	42	4	4
Non ICU	2	80	18
ICU	3	27	95

Figure S3. Discriminant analysis performed on the selected variables according to top SHAP values in Fig. 5 (c) considering Control, Admitted to ICU, and not admitted to ICU.

A

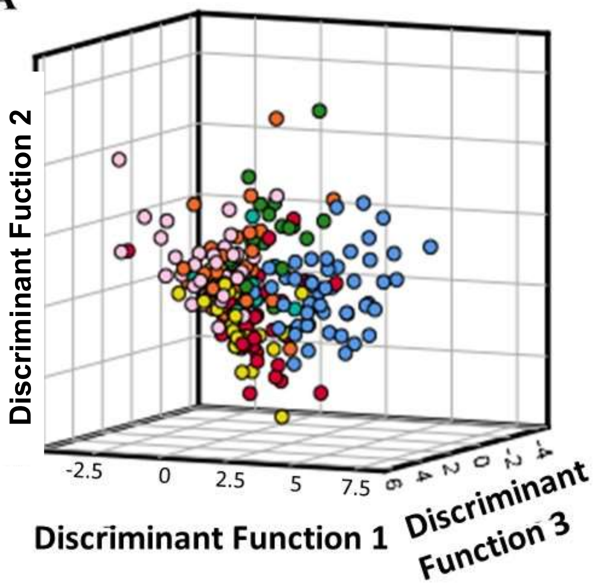

1	*Control*	
2	No ICU	> 65 no ICU
3	Post ICU	> 65 post_ICU
4	ICU	> 65 ICU
5	No ICU	< 65 no ICU
6	Post ICU	< 65 post_ICU
7	ICU	< 65 ICU

Confusing Table case A. Cross validation 55.3 %

Class	Predicted Class						
	1	*2*	*3*	*4*	*5*	*6*	*7*
1	*42*	3	1	0	0	4	0
2	1	*31*	2	3	8	2	3
3	0	1	*11*	7	0	3	0
4	0	6	5	*19*	8	3	9
5	1	14	0	8	*23*	2	2
6	1	1	2	1	0	*3*	3
7	0	2	1	6	4	6	*23*

B

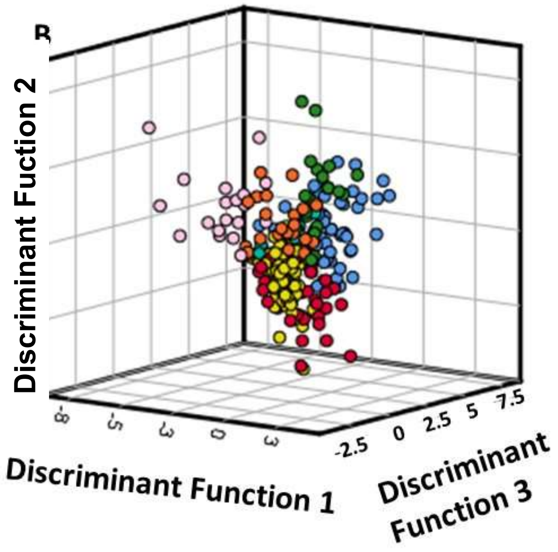

1	*Control*	
2	No ICU	>65 no ICU
3	Post ICU	>65 post_ICU
4	ICU	>65 ICU
5	No ICU	<65 no ICU
6	Post ICU	<65 post_ICU
7	ICU	<65 ICU

Confusing Table case B. Cross validation 50.5 %

Class	Predicted Class						
	1	*2*	*3*	*4*	*5*	*6*	*7*
1	*44*	4	0	0	1	1	0
2	3	*17*	2	2	25	0	1
3	1	1	*10*	3	4	3	0
4	1	4	5	*12*	24	1	3
5	1	7	1	2	*38*	1	0
6	1	0	2	3	3	*2*	0
7	2	4	0	8	11	1	*16*

Figure S4. Discriminant Analysis results considering A: all m/z values and B: Shapley variables selected.
